# Multiparametric Evaluation of Head and Neck Squamous Cell Carcinoma Using a Single-Source Dual-Energy CT with Fast kVp Switching: State of the Art

**DOI:** 10.3390/cancers7040886

**Published:** 2015-11-06

**Authors:** Stephanie Lam, Rajiv Gupta, Hillary Kelly, Hugh D. Curtin, Reza Forghani

**Affiliations:** 1 Department of Radiology, Jewish General Hospital, McGill University, Montreal, QC H3T 1E2, Canada; stephanie.lam@mail.mcgill.ca; 2 Department of Radiology, Massachusetts General Hospital, Harvard Medical School, Boston, MA 02114, USA; rgupta1@mgh.harvard.edu (R.G.); hillary.kelly@mgh.harvard.edu (H.K.); 3 Department of Radiology, Massachusetts Eye and Ear Infirmary, Harvard Medical School, Boston, MA 02114, USA; hugh_curtin@meei.harvard.edu; 4 Lady Davis Research Institute, Montreal, QC H3T 1E2, Canada; 5 Segal Cancer Centre, Jewish General Hospital, McGill University, Montreal, QC H3T 1E2, Canada

**Keywords:** head and neck squamous cell carcinoma, dual energy CT, fast kVp switching, virtual monochromatic image, iodine overlay map, thyroid cartilage invasion, dental artifact reduction, lymphadenopathy

## Abstract

There is an increasing body of evidence establishing the advantages of dual-energy CT (DECT) for evaluation of head and neck squamous cell carcinoma (HNSCC). Focusing on a single-source DECT system with fast kVp switching, we will review the principles behind DECT and associated post-processing steps that make this technology especially suitable for HNSCC evaluation and staging. The article will review current applications of DECT for evaluation of HNSCC including use of different reconstructions to improve tumor conspicuity, tumor-normal soft tissue interface, accuracy of invasion of critical structures such as thyroid cartilage, and reduce dental artifact. We will provide a practical approach for DECT implementation into routine clinical use and a multi-parametric approach for scan interpretation based on the experience at our institution. The article will conclude with a brief overview of potential future applications of the technique.

## 1. Introduction

Although there has been interest in dual-energy computed tomography (DECT) since the 1970s, it is not until recent technological advances permitting almost simultaneous image acquisition at two different energy levels in a single scan that DECT has become possible for routine clinical use. Since the introduction of clinical DECT scanners in 2006 [1,2,3], there has been increasing literature on potential applications of DECT in all major subspecialties in radiology [4,5,6,7]. In particular, there is increasing evidence supporting the advantages of using DECT for evaluating head and neck pathology, with specific applications for head and neck squamous cell carcinoma (HNSCC) [8,9,10,11,12,13,14,15,16,17].

Squamous cell carcinoma is the most common malignancy of the head and neck, other than non-melanoma skin cancers [18]. Conventional, single-energy CT (SECT) is often the first line imaging modality used for characterization, staging, and follow-up of HNSCC below the level of the hard palate [11,13,19,20]. Because of the often infiltrative growth of HNSCC and the potential for image degradation secondary to various artifacts, achieving images with high lesion contrast, high image quality, and low noise, is essential for optimal delineation of lesions from surrounding tissue and consequently accurate tumor staging and treatment planning [11]. While the technical innovations of multidetector CT (MDCT) have led to substantial improvements in image quality in the last decade, the advent of DECT and its unique post-processing capabilities has now made possible additional tissue characterization and image processing beyond what is possible with conventional, SECT scanners [6,11,15].

In this article, we will review the basic principles behind DECT and the use of DECT reconstructions for improving HNSCC evaluation and staging, focusing on a single-source DECT system with fast kVp switching. We will review the use of different DECT reconstructions for enhancing tumor conspicuity, tumor interface with surrounding normal soft tissues, accuracy of invasion of critical structures such as thyroid cartilage, and dental artifact reduction. We will also provide a practical, multi-parametric approach for scan interpretation and implementation into clinical use based on current evidence and the experience at our institution. Finally, we will provide a brief overview of advanced DECT analysis and other potential future applications for head and neck cancer evaluation.

## 2. Basic Principles of DECT

Conventional, SECT uses X-rays generated from a rotating tube at a single fixed potential to expose digital detectors after passing through and being attenuated by the patient. The signal that is detected reflects the intensity of the X-ray after attenuation through the patient. Thus, CT images are pictorial representations of the relative attenuation values of tissues, quantified in Hounsfield units (HU) [6,21,22,23].

Tissue attenuation depends not only on the energy spectrum of the x-ray beam, but also on the density and elemental composition of the material through which it passes [22,23]. While images obtained with SECT provide excellent structural information, material-specific information can sometimes be limited. At a fixed tube potential, different materials can have the same attenuation, and consequently be difficult to distinguish [5].

With DECT, the same anatomic structure is imaged at two different peak energy levels, typically 140 kVp and 80 kVp, providing high and low energy spectra for imaging [3]. The two resulting image datasets allow for analysis of energy-dependent changes in attenuation of different materials, enabling spectral evaluation of tissues at different energy levels [3,5]. Since certain materials or tissues may attenuate x-ray beams differently at different energy levels, spectral evaluation using DECT may be used to accentuate clinically relevant characteristics of the tissue of interest, or improve differentiation of certain tissues. Spectral characterization using DECT is highly dependent on the atomic number (Z) of tissues. For example, many common constituents of normal tissues in the human body have low atomic numbers and would be difficult to distinguish based on their spectral properties. However, iodine has a much higher atomic number (Z = 53), which results in a strong spectral contrast between the heavy atoms of iodinated contrast agents used in CT scanning, and the light atoms of the tissues in the body [3,24]. This property can be exploited to improve evaluation of enhancing lesions such as HNSCC, as will be described and illustrated later in this article.

## 3. Types of DECT Scanners

**Figure 1:**
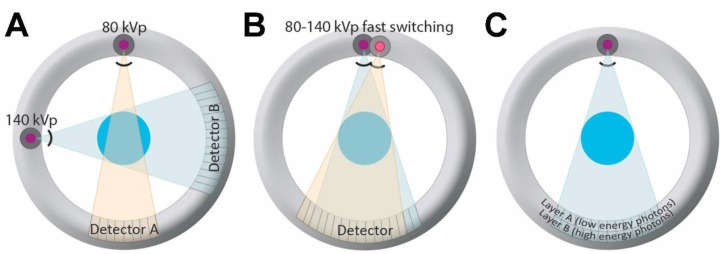
Different dual-energy CT (DECT) scanners currently in clinical use. (**A**) Illustration of a dual source DECT, consisting of two source x-ray tubes with corresponding detectors; (**B**) Illustration of a single source DECT with rapid kVp switching. With this type of scanner, the tube voltage follows a pulsed curve, and projection data are collected twice for every projection, one at high and one at low tube voltage, during rapid kVp switching; (**C**) Illustration of a dual layer DECT, consisting of a single source and single (but layered) detector. The detector is composed of two scintillation layers enabling separation of high and low energy spectra produced by a single source.

There are currently three major types of DECT scanners that use different techniques to acquire high energy and low energy datasets: a dual-source dual-energy scanner, a single-source dual-energy scanner with fast kilovoltage peak (kVp) switching, and a single-source dual-energy scanner with a dual-layer detector (Figure 1).

A dual-source DECT scanner uses two x-ray tubes with corresponding detectors aligned at a 90° angle (Figure 1). Each x-ray tube operates at a different potential, one at higher energy, and the other at lower energy (typically 140 kVp, and 80 kVp, respectively) (Siemens AG, Forchheim, Germany) [3,25]. A single-source DECT scanner with fast kVp switching uses a single X-ray tube and detector combination. With this technique, the tube voltage follows a pulsed curve, and projection data is collected twice for every projection, one at high and one at low tube voltage, during rapid kilovoltage switching at the X-ray source (GE Healthcare, Milwaukee, WI, USA) [3,26]. A single-source dual-energy scanner with a dual-layer detector uses a single source and a single detector, but the detector has two layers that are used to distinguish between high and low energy photons (Philips Healthcare, Andover, MA, USA) [3,5,6,22,27,28]. Recently, another type of single source DECT system has been introduced in which the x-ray beam from a single tube is pre-filtered and split into high and low energy spectra before reaching the patient (TwinBeam Dual Energy CT; Siemens AG). There are also other modes of DECT acquisition possible, including sequential acquisition, although this does not have as good a temporal resolution and therefore may not be optimal for evaluation of enhancing lesions that can have changes in attenuation over time. A more detailed technical discussion of DECT is beyond the scope of this article but can be found elsewhere [29].

## 4. Radiation Dose Considerations

The potential harmful effects of radiation exposure from occasionally performed diagnostic imaging studies is debatable [30,31], particularly in the adult head and neck cancer population, who may undergo radiation therapy as a part of their treatment. Nonetheless, radiation exposure from medical imaging must always be considered, and the radiation dose should be as low as reasonably achievable while obtaining scans of high diagnostic quality. It should also be noted that image quality and radiation exposure from a CT scan are inter-related. Therefore, any comparison of dose made between different acquisition techniques must also take into account effects on image quality.

Early after the introduction of clinical DECT scanners, concerns were raised regarding increased radiation exposure to the patient, reported as up to three times compared to a conventional SECT acquisition [32]. However, subsequent improvements in acquisition and post processing techniques have largely overcome these concerns. An initial study in 2007 by Johnson *et al.* [2] that primarily evaluated material differentiation, demonstrated radiation doses well below reference dose values for the respective body regions. A subsequent study performed on phantoms by Schenzle *et al.* [33] compared the radiation doses of first- and second-generation dual-source systems to conventional CT, using different pulmonary CT angiography protocols. The authors found that dose was similar between DECT and SECT protocols, and that lower contrast-to-noise (CNR) ratio with one of the DECT protocols could be overcome, and even improved compared to SECT, by using blended images. More recent studies evaluating various anatomic regions have shown that DECT may even result in a lower radiation dose than SECT, while maintaining similar image quality [33]. These improvements have been attributed to advances in CT technology and software [34].

One of the earliest studies using a single-source DECT scanner with fast-kVp switching considered radiation dose in abdominal imaging protocols. Longer gantry revolution time on first-generation software resulted in significantly higher radiation doses than those of conventional SECT [32]. However, more recent studies with this type of scanner have shown radiation dose levels closer or similar to those of SECT. Using this DECT technique, Li *et al.* [35] reported 14% higher dose for DECT compared to SECT for a body exam or 22% higher dose for an examination of the head. However, Zhang *et al.* [36] observed that at equal doses, the diagnostic performance of DECT and SECT were equivalent in abdominal imaging.

Tawfik *et al.* [37] directly evaluated radiation doses in the head and neck using a dual-source scanner. They reported that both quantitatively and qualitatively, image quality of DECT was comparable to that of SECT, but with a 12% lower radiation dose. Since then, other studies evaluating the head and neck, while not primarily studying radiation dose, have also stated that DECT radiation dose was similar to or less than that of SECT [13]. At our institutions using fast kVp switching scanners, we also routinely obtain neck CTs in dual energy mode without a significant dose penalty compared to SECT scans.

DECT enables multiple additional reconstructions through post-processing that are not possible with SECT. Therefore, if the DECT acquisition can be performed with a similar dose and at least equivalent quality to a SECT, then any additional information obtained from other DECT-specific reconstructions or analysis would represent a relative advantage compared to SECT, since this additional information is essentially obtained for “free”. In addition, because DECT can be used to create virtual unenhanced images for detection of stones, or iodine overlay maps providing a quantitative estimate of tissue iodine content, DECT may under some circumstances result in a reduced number of acquisitions needed as compared to SECT. For example, in abdominal imaging, generation of virtual unenhanced images using DECT post-processing techniques can eliminate the need to perform a true unenhanced acquisition in some CT protocols. As a result, radiation exposure is significantly reduced, which is an additional potential advantage of DECT relative to SECT, for carefully selected applications [38].

## 5. Improved HNSCC Evaluation and Soft Tissue Boundary Delineation Using Low Energy Reconstructions

CT is often the first line imaging modality for initial staging and follow-up of most HNSCC. Unless contraindicated, CT scans of the neck performed for evaluation of head and neck cancer should be performed after administration of intravenous contrast. Contrast is administered in order to improve soft tissue contrast and to help distinguish tumor from normal tissues or vital structures such as vessels. Administration of iodinated CT contrast agents improves detection and delineation of tumors because of differences in tumor vascularity and enhancement patterns compared to normal soft tissues. However, even on contrast-enhanced scans, tumor can have similar attenuation to certain normal tissues, such as muscle and non-ossified thyroid cartilage, and at times making a distinction can be challenging [16,19,39,40].

When a DECT scan is obtained, one type of image that can be generated is the virtual monochromatic image (VMI). These images represent simulated tissue attenuation as it would appear if the patient was imaged with a monochromatic X-ray beam at the specified energy level. When imaging with a single-source dual-energy CT scanner with fast kVp switching, data can be processed to obtain images at any energy level between 40 and 140 kiloelectron volts (keV) [26]. With a dual source type scanner, images may also be reconstructed at energy levels higher than 140 keV.

The main physical interactions at X-ray energies being considered (60–150 keV), and those required for spectral tissue characterization, are the photoelectric effect and Compton scattering. However, spectral tissue characterization is particularly dependent on the photoelectric effect. The photoelectric effect is highest just above the k-edge of an atom, which represents the binding energy of the innermost, or K-shell, electrons. The k-edge of iodine is 33.2 keV [3]. As such, the conspicuity or attenuation/density of iodine containing tissues such as enhancing tumor becomes higher on lower energy VMIs approaching the iodine k-edge. However, this comes at the expense of increased image noise on lower keV VMIs. The reason for this is an increased proportion of scatter relative to signal generating photons at lower energies, and this trade-off needs to be taken into consideration.

**Figure 2:**
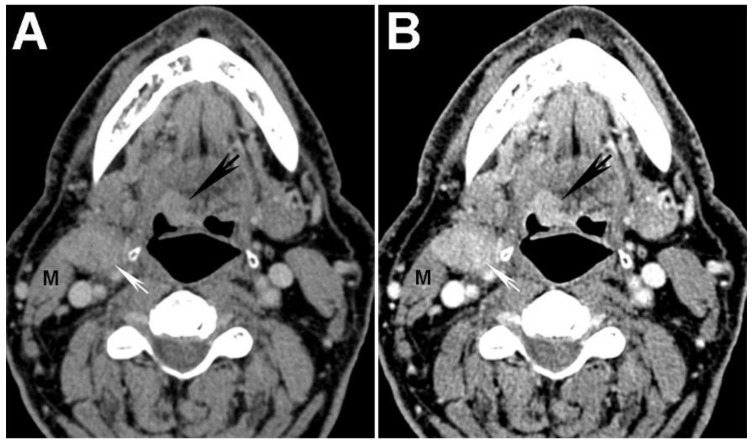
Increased tumor attenuation on 40 keV virtual monochromatic images (VMIs). (**A**) 70 keV single energy equivalent CT image of a right base of tongue tumor (large black arrow) and pathologic right level IIA lymph node (small white arrow) is shown. Note the similar density of both lesions compared to the normal right sternocleidomastoid muscle (M); (**B**) On the 40 keV image displayed using the same window-level settings, note the higher lesion density as well as higher relative contrast compared to muscle (M). Also note the increased image noise on the 40 keV VMI (**B**) compared to 70 keV VMI (**A**).

**Figure 3:**
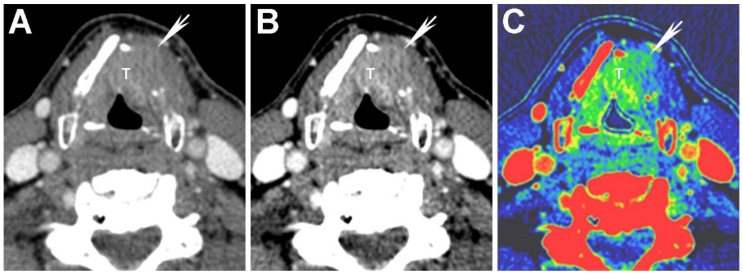
Virtual monochromatic images (VMIs) and iodine overlay map of a laryngeal tumor (T) invading the left thyroid cartilage. (**A**) 65 keV VMI; (**B**) 40 keV VMI; and (**C**) iodine-water (iodine overlay) material decomposition maps are shown. Note the increased tumor conspicuity on the 40 keV (**B**) compared to the 65 keV (**A**) VMIs. The iodine overlay map provides a quantitative estimate of iodine content of different tissues and demonstrates iodine containing tumor transgressing the left thyroid cartilage (arrow). It is noteworthy that the tumor edge of the extralaryngeal component (arrow) is more clearly seen on the 40 keV VMI (**B**) and iodine overlay map (**C**) than on the SECT equivalent 65 keV VMI (A).

The increased attenuation of iodine at lower energies can be taken advantage of in order to increase the visual conspicuity and measured attenuation/density of tumor using DECT. In a study performed using a single-source DECT scanner with fast kVp switching, Lam *et al.* [17] evaluated optimal VMI reconstructions for assessment of normal anatomy and HNSCC. The authors concluded through objective analysis of signal-to-noise ratio (SNR) that 65 keV VMIs had the best overall image quality and suggested that it should be used as the default reconstruction for assessment of the neck. The optimal, highest SNR at 65 keV was closely followed by the 70 keV VMIs, the typical default reconstruction believed to most closely resemble the standard 120-kVp SECT acquisition, based on data extrapolated from body imaging [41,42]. In the same study, it was also shown that tumor conspicuity/attenuation is highest on 40 keV VMIs [17] (Figure 2 and Figure 3). Furthermore, it was shown that the attenuation difference between tumor and muscles was also highest on the 40 keV VMIs, despite the higher image noise on these reconstructions [17] (Figure 2 and Figure 3). We have recently expanded on these observations using data obtained at our two different institutions and also demonstrated that subjectively, 40 keV is the preferred VMI for targeted tumor evaluation by both general radiologists and those who specialize in head and neck imaging [43].

Higher tumor attenuation on 40 keV VMIs has also been reported using a dual-source DECT scanner [15,44]. However, at least in one study by Wichmann *et al.*, the 60 keV VMIs, rather than the 40 keV VMIs were reported as having the highest tumor contrast to noise ratio and subjective overall image quality [15]. At this time, it is not clear whether the discrepancy between optimal VMI energy level described in the study by Lam *et al.*, using a fast kVp switching scanner, and the study by Wichmann *et al.*, using a dual source scanner, is secondary to technical differences between the scanners or secondary to differences in image acquisition (the acquisition dose was lower in the study by Wichmann *et al.*). In a later study by Albrecht *et al.*, it was reported that by creating an altered or “advanced” form of monoenergetic reconstructions, the objective contrast to noise ratio of tumors was highest on the “advanced” 40 keV images, but the 55 keV images were preferred subjectively [44]. The algorithm used for generation of “advanced” 40 keV reconstructions combined the high signal data obtained at low energies with the superior noise properties seen at medium energies into one reconstructed image. In addition to using low energy VMIs to improve tumor visualization, other studies have reported using different weighting or blending factors for WA images to improve tumor conspicuity [11]. At this time, there is not yet consensus on the optimal reconstructions for tumor evaluation using a dual-source scanner. There are currently no published reports evaluating HNSCC using a dual layer scanner type.

## 6. Evaluation of Thyroid Cartilage Invasion

Accurate determination of thyroid cartilage invasion is important for proper staging and consequently management and treatment of laryngeal and hypopharyngeal tumors [8,19,45,46]. Both CT and MRI can be used to evaluate cartilage invasion, and each imaging modality has advantages and limitations for staging of laryngeal and hypopharyngeal cancer [19,39,47]. Using a combination of specific diagnostic criteria (sclerosis, erosion, lysis, and extralaryngeal spread) for the evaluation of laryngeal cartilages, Becker *et al.* have reported that CT can have an overall sensitivity of 82%, specificity of 78%, and negative predictive value of 91% [20,39]. When focusing on thyroid cartilage, sensitivity and specificity were 71% and 83% respectively [48]. MRI can also be used to evaluate thyroid cartilage invasion and is reported to have a very high negative predictive value (94%–96%), but a lower specificity (74%–88%). When considering invasion of the thyroid cartilage specifically, at least some studies suggest that there is a greater potential for false positives on MRI because of reactive inflammation, edema and fibrosis that may mimic invasion by tumor [20,39]. Obtaining high diagnostic quality MRI examinations can also be problematic in the head and neck cancer population who may have difficulty clearing their secretions and remaining motionless during the much longer examination times for this modality compared to CT.

DECT acquisitions enable creation of reconstructions based on two material or even three material decomposition [3,24]. These reconstructions can be used to isolate materials of interest relative to a reference material, assuming that the two materials have sufficiently different spectral properties. Two common types of reconstructions generated using material decomposition are maps reflecting the iodine content of different tissues, called iodine overlay maps, and virtual unenhanced images [3,24,38]. Iodine overlay maps are of interest in the assessment of HNSCC since they provide a quantitative estimate and a visual representation of the iodine content of different tissues, such as enhancing tumor (Figure 3).

In a study performed using a dual-source DECT, Kuno *et al.* [9] used iodine maps to help differentiate areas of tumor invasion from normal laryngeal cartilage (Figure 3). The authors showed that the addition of iodine overlay maps to the weighted-average (WA) equivalent of standard SECT 120-kVp images resulted in increased specificity (96% *vs.* 70%) for the diagnosis of invasion of laryngeal cartilage, without compromising the sensitivity (86% *vs.* 86%). These values show an improvement over those previously reported in the literature (83% specificity and 71% sensitivity) [48]. Furthermore, Kuno *et al.* also reported improved inter-observer reproducibility with the addition of iodine maps. Improved specificity and inter-observer reproducibility result in reduced overestimation of tumor invasion, and may help reduce unnecessary laryngectomies [9,46].

**Figure 4:**
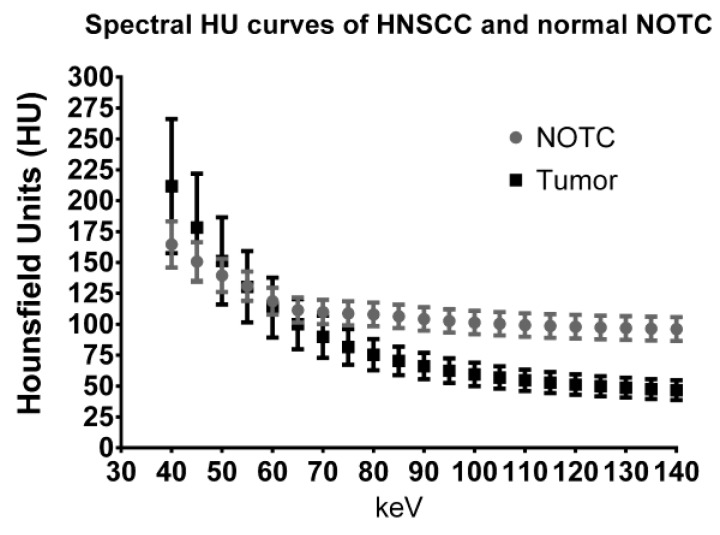
Quantitative region of interest analysis comparing the spectral Hounsfield unit attenuation curves of HNSCC to non-ossified thyroid cartilage (NOTC) [16]. Pooled analysis of 30 tumors and NOTC from 30 normal patients is shown. At 65 or 70 keV, the attenuation of tumor can be very similar to normal NOTC, explaining the difficulties that may be encountered in differentiating between the 2 on conventional single energy CT scans. On the other hand, there is spectral separation at either end of the curve, with the best density separation achieved in the high energy range.

**Figure 5:**
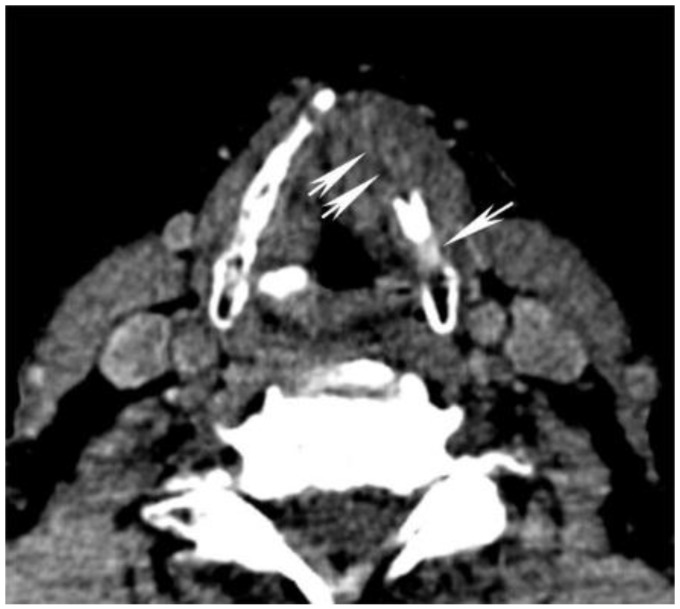
High energy virtual monochromatic images (VMIs) for evaluation of non-ossified thyroid cartilage (NOTC). 140 keV image from the same patient as in Figure 3 is shown. The laryngeal tumor invades the left thyroid cartilage, and the invaded portion appears as a relatively low density defect (double arrows) because of suppression of iodine density within the enhancing tumor on high keV images (compare to Figure 3A,B). In this case, there is partial non-ossification of the thyroid cartilage on the left posteriorly. Note the preserved high attenuation of the NOTC (single arrow). There is clear attenuation difference between normal NOTC and tumor on the 140 keV image but the density on conventional single energy equivalent 65 keV image is nearly identical (compare to Figure 3A). It is noteworthy that the tumor itself is not well seen on the 140 keV images, and these VMIs should be used in conjunction with the 65 and/or 40 keV VMIs and not in isolation.

Another type of reconstruction that may be useful for evaluating thyroid cartilage invasion is the high energy VMI. Although the attenuation of tumor is very different from that of ossified thyroid cartilage (cortical bone and bone marrow), attenuation of tumor can be similar to that of non-ossified thyroid cartilage (NOTC) [8,16,19,39,40,49,50]. This similarity in attenuation can make detection of small or early invasion problematic in cases where tumor abuts NOTC. In a study using a single-source DECT with fast kVp switching, Forghani *et al.* [16] compared the spectral attenuation values of HNSCC to normal NOTC. Although this study did not directly evaluate thyroid cartilage invasion, the authors demonstrated that the spectral Hounsfield unit attenuation curves of tumor and NOTC are different, especially at high energies (Figure 4). Specifically, there was progressive separation of the attenuation curves of tumor and NOTC at high energies, without overlap of the curves on VMIs of 95 keV or higher in that patient population [16]. The findings suggest that high energy VMIs may also be helpful for evaluating thyroid cartilage invasion. Based on these observations, tumor invading cartilage would appear as a relatively low density gap, whereas normal NOTC would preserve a relatively high attenuation on the high energy reconstructions (Figure 5). It is noteworthy that since tumor attenuation is suppressed on high energy VMIs, these should be used in conjunction with the 65 and 40 keV VMIs. High energy VMIs are not meant to be used in isolation.

## 7. Dental Artifact

There is a high prevalence of dental restorations in the general and HNSCC patient population and depending on the type and amount of dental material present, there can be significant associated artifact and image degradation. This can in turn reduce the diagnostic accuracy of exams and obscure lesions such as tumors, particularly in the oral cavity but also to a lesser extent at other sites, including in the oropharynx. High-density objects like metal create artifact through several mechanisms: photon starvation, beam hardening, and scatter [51,52]. Because of the high density of metal, the entire spectrum of the X-ray beam is absorbed, so-called photon starvation, resulting in zero-transmission, which complicates the filtered back-projection. Beam hardening occurs because of the polychromatic nature of the X-ray beam. As the beam passes through the tissue, the lower energy photons are absorbed disproportionately as compared to the higher energy photons. The X-ray beam that is detected therefore contains photons from the higher energy portion of the spectrum, which manifests as dark streaks [51,52,53,54,55]. Scatter results from the high x-ray attenuation coefficient of bone and metal [51].

Though the extent of artifact can be a function of factors intrinsic to the object causing the artifact, such as shape, size and material composition, modifying the acquisition parameters of the CT scan can reduce artifact [56]. Increasing the peak voltage and the tube current, narrowing the collimation, and optimizing image reconstruction are ways to reduce artifact [51,57,58]. However, the application of these methods may result in a greater radiation exposure or decreased spatial resolution [58]. Certain interpolation techniques may even result in the creation of new artifacts [52,53]. Iterative reconstruction algorithms or sinogram inpainting methods have also been shown to reduce metallic artifact, but not all of these techniques are commercially available for routine use in clinical practice, at least in part because of the large computational power required for some of the algorithms [6,57,59,60].

**Figure 6:**
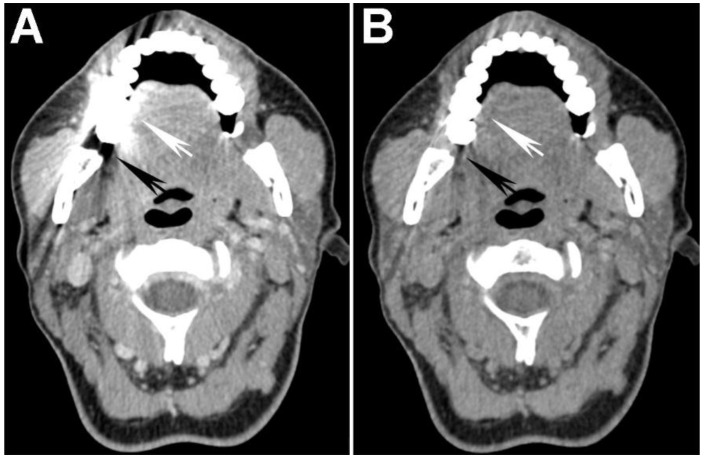
Use of high energy DECT virtual monochromatic images (VMIs) for dental artifact reduction. (**A**) 65 keV and (**B**) 140 keV VMIs are shown from the same level in the neck. Note significant reduction of artifact such as in the region of retromolar trigone (black arrow) or oral tongue (white arrow) on the higher energy, 140 keV VMI compared to the 65 keV VMI.

**Figure 7:**
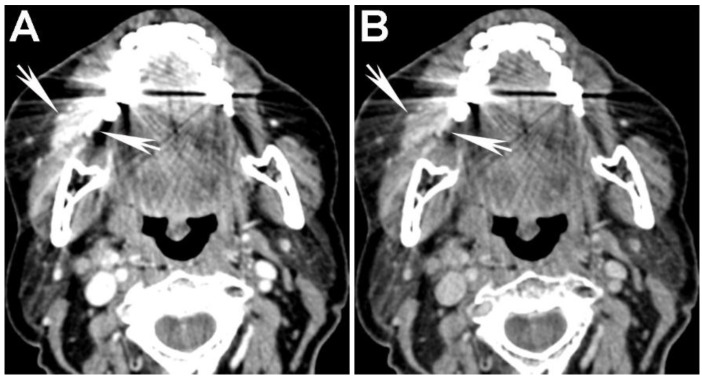
Use of high energy DECT virtual monochromatic images (VMIs) for dental artifact reduction in a patient with a gingival-buccal tumor. (**A**) 65 keV (equivalent to single energy CT) and (**B**) 95 keV VMIs are shown from the same level in the neck. Note reduction of artifact and improved visualization of the enhancing tumor on the 95 keV VMI compared to the 65 keV VMI.

In clinical practice, imaging at higher peak voltage reduces the magnitude of artifacts, and may potentially represent an approach to improving the quality of CT imaging in patients with dental hardware. Using DECT to reconstruct VMIs that simulate imaging at higher energy levels may therefore reduce metallic artifact [53] (Figure 6 and Figure 7). This effect has been investigated in a number of *in vivo* and *ex vivo* studies using orthopedic hardware, and high energy VMIs have been shown to reduce metallic artifact significantly, both quantitatively and qualitatively, improving assessment of the implant, the surrounding bone, and soft tissue interface [51,52,53,56,57,58,61].

There has been promising research investigating dental hardware artifact reduction. A study conducted by Stolzmann *et al.* [55] in cadavers with dental restorations and bridges (but no implants or full dentures) using a dual-source DECT scanner evaluated artifact reduction at high energy VMIs and compared the results to conventional SECT. The study showed that the extent of artifacts decreased as the VMI energy level increased, particularly at energy levels greater than or equal to 88 keV, and that there was less artifact on VMIs than on standard SECT images. Overall, through reduction of metallic artifact, image quality was improved on VMIs compared to SECT. Qualitative evaluation was in agreement with the quantitative assessment.

In a study performed on a dual-source DECT in patients with dental implants, Tanaka *et al.* [62] compared VMIs at 100 keV to VMIs at 190 keV to fused DECT images corresponding to the standard SECT 120 kVp acquisition. In this study, 100 keV VMIs were superior to both 190 keV VMIs and fused 120 kV images, for the reduction of dark bands and consequently, for the evaluation of adjacent bone.

Most recently, De Crop *et al.* [60], in a cadaveric and phantom study, evaluated different artifact reduction techniques compared to SECT. The authors concluded that overall, model-based iterative reconstruction was most promising, with significant improvement of image quality without loss of image contrast. High energy VMIs (140 keV) obtained with a DECT scanner using fast kVp switching also showed good reduction of artifact, but with lower image contrast at higher energy levels. The authors found that the concomitant use of metal artifact reduction software actually decreased image contrast, negatively affecting image quality. The study also evaluated different materials used in dental hardware, confirming that titanium caused the least amount of artifact on CT, with cobalt-chromium contributing slightly more, and zirconium the most.

There is not yet consensus on the optimal VMI energy level for reduction of dental artifact. Studies evaluating orthopedic hardware have found the optimal VMI to be greater than 90–110 keV on single-source DECT [51,57,61], while optimal VMI on dual-source DECT was between 95 and 150 keV [52,53,56,58]. Pilot studies evaluating VMIs for reduction of dental artifact show similar values, with reported energy levels greater than 88–100 keV for dual-source DECT [55,62], and 140 keV for single-source DECT using fast kVp switching. One of the challenges in dealing with dental artifact is that there can be significant variation depending on the type and amount of material used. The other variable that must be considered is the target lesion being evaluated. For example, if the objective is to better assess bone detail (or invasion) in an area obscured by artifact, there may be much greater leeway or benefit for significantly higher energy VMIs, approaching or even exceeding 140 keV. However, if the objective is to better visualize the actual enhancing tumor, then more intermediate energy VMIs may have to be considered that balance the increased artifact reduction with decreased tumor attenuation and contrast on higher energy VMIs (Figure 7). This is currently being investigated by our group. Nonetheless, so far the evidence shows that high energy DECT VMIs are promising and have the potential for improving lesion assessment in areas obscured by artifact, such as the oral cavity. Further refinements in applications of DECT for artifact reduction, and how they compare to other methods that are being developed using SECT [60,63], are an interesting topic for future research.

## 8. Other Potential DECT Applications for the Evaluation of HNSCC

There are a few studies suggesting that DECT can improve evaluation of pathologic lymph nodes. In the study by Lam *et al.* [17], it was demonstrated that in addition to improving visibility of the primary lesion, 40 keV VMIs also improve conspicuity of metastatic lymph nodes. We are currently investigating potential use of these reconstructions for evaluation of nodal heterogeneity. Tawfik *et al.* [12] have reported that contrast content of metastatic HNSCC lymph nodes is less than that of normal lymph nodes, as quantified by iodine content calculation through the use of iodine overlay maps produced by DECT. Liang *et al.* [64] have also shown that there can differences in spectral characteristics of metastatic lymph nodes compared to non-metastatic lymph nodes. In their study, they evaluated the slope of lymph node attenuation with respect to the energy level, and calculated the ratio relative to the primary lesion. They reported that the ratio of the slopes was greater for metastatic lymph nodes than for normal lymph nodes, which may not be in agreement with observations from Tawfik’s group. Optimal evaluation of lymph nodes using DECT requires further investigation and is an interesting topic for future research.

## 9. Practical, Multi-Parametric DECT Approach for HNSCC Evaluation

One of the major advantages of imaging with DECT is that through sophisticated post-processing, different reconstructions can be generated without the need for additional patient scanning or radiation exposure. Although one approach may be to try to find a single best reconstruction that captures all the clinically relevant information, it may not be possible to do so because there is often a trade-off between different key parameters of interest. For example, low energy reconstructions improve lesion conspicuity but at the expense of image noise. High energy reconstructions appear to improve evaluation of thyroid cartilage and reduce dental artifact, but suppress iodine density and therefore result in decreased visibility of the tumor itself. Iodine overlay maps also contain important information regarding the iodine content of the tumor and have been shown to improve accuracy for determination of thyroid cartilage invasion, but lack the complete anatomic information that is available on standard CT reconstructions.

Therefore, instead of relying on a single set of reconstructions, based on our experience and current literature, we recommend a multi-parametric approach to HNSCC evaluation using DECT. Our recommended approach is to supplement the standard single energy CT equivalent VMIs with additional reconstructions targeted for evaluation of specific characteristics of clinical interest, similar to the use of different MRI sequences for lesion characterization. Based on our experience, the high SNR 65 (or 70) keV VMIs should be used for standard evaluation of the neck and as reference images for normal anatomy. These should be supplemented with low energy (40 keV) VMIs for targeted evaluation of the tumor and tumor-soft tissue boundary in all HNSCC cases. For laryngeal and hypopharyngeal tumors, we also recommend complementary evaluation with high energy VMIs (95 keV or greater) and iodine overlay maps, in order to increase accuracy for evaluation of thyroid cartilage invasion. Lastly, the use of high energy VMIs should also be considered for evaluation of oral cavity and possibly oropharyngeal tumors, in order to reduce dental artifact. However, the optimal energy for dental artifact reduction in tumor containing areas is yet to be determined. These recommendations are for a single energy DECT with fast kVp switching and are summarized in Table 1. It is our opinion that the same overall approach is likely to be beneficial using a dual source scanner (and presumably a dual layer scanner), although there is no clear consensus at this time on the exact reconstruction energies that are best for a dual source scanner.

**Table 1:** Recommended^†^ multi-parametric approach for the evaluation of head and neck squamous cell carcinoma using a single source fast kVp switching dual energy CT scanner.

**Recommended reconstructions for all dual energy CT scans of the neck:**
65 and 40 keV VMIs
**Recommended reconstructions for evaluation of laryngeal tumors:**
65 and 40 keV VMIs + Iodine overlay maps; High energy VMIs (95 keV or greater)
**Recommended reconstructions for evaluation of oral cavity and possibly oropharyngeal tumors:**
65 and 40 keV VMIs + Consider supplemental high energy VMIs for dental artifact reduction

^†^ Recommendations are based on the current published evidence and the practice at the author’s institutions and are not meant to be exclusive or all encompassing. These are based on a small number of studies and it is likely that there is room for additions or small adjustments according to specific institutional protocols and preferences.

## 10. Future Prospects and Concluding Remarks

Dual energy CT spectral tissue characterization is an exciting and active area of research. It is likely that additional refinements in technique and recommendations for use of different reconstructions for HNSCC evaluation will emerge for the different DECT platforms in the near future. One of the important considerations for routine clinical use is workflow friendly implementation. Depending on the scanner type and version of the console, different energy VMIs and iodine overlay maps can be generated by the technologists and sent to PACS for clinical use. Some of the newer consoles for certain types of scanners also enable automatic generation of both high and low energy VMIs. Furthermore, some DECT platforms have post-processing software integrated with the clinical PACS, allowing workflow friendly integration. These should facilitate and increase the use of this exciting technology. Implicit in this set up is increased use of resources, including technologist and potentially radiologist time, as well informatics resources such as PACS/IT storage. Therefore, widespread and continuing use of DECT and its spectral capabilities is contingent on the additional value provided for the diagnostic work up and treatment planning for our patients. As such, continued research in this area is of paramount importance, in order to further advance and demonstrate the potential additional value of DECT for tumor characterization. In this regard, in addition to what has been discussed in this article, spectral data from DECT scans contain a wealth of quantitative information that could be potentially be utilized for more advanced tissue and image analysis and these represent exciting topics for future investigations in the era of personalized medicine.
